# Sperm DNA fragmentation and male age: results of *in vitro* fertilization treatments

**DOI:** 10.5935/1518-0557.20210018

**Published:** 2021

**Authors:** Estefanía Martínez, Constanza Bezazián, Ana Bezazián, Karen Lindl, Anabela Peliquero, Antonio Cattaneo, Diego Gnocchi, Marcela Irigoyen, Lautaro Tessari, A. Gustavo Martínez

**Affiliations:** 1 Fertilis Reproductive Medicine, San Isidro, Buenos Aires, Argentina; 2 Department of Biology, School of Sciences, Belgrano University, Buenos Aires, Argentina

**Keywords:** assisted reproduction, sperm DNA fragmentation, male age, miscarriage rate

## Abstract

**Objective:**

This study aimed to assess the effects of sperm DNA fragmentation in parents belonging to different age groups. The couples included in the study comprised normozoospermic men and infertile women undergoing conventional IVF.

**Methods:**

The results obtained from 163 conventional IVF cycles were analyzed retrospectively. The couples enrolled in the study included women aged between 30 and 37 years. Sperm DNA fragmentation was studied using the TUNEL assay. The patients were split into four groups based on male age and sperm DNA fragmentation, as follows: Group 1: ≤39 years and TUNEL assay ≤20%; Group 2: ≤39 years and TUNEL assay >20%; Group 3: ≥40 years and TUNEL assay ≤20%; and Group 4: ≥40 years and TUNEL assay >20%.

**Results:**

No significant differences were found in semen parameters or fertilization rates between groups. Groups with <20% sperm DNA fragmentation showed significant differences in other parameters, including higher blastocyst formation rate (Group 1: 63% and Group 3: 60% vs. Group 2: 43% and Group 4: 41%, *p*<0.05) and higher expanded blastocyst formation rate (Group 1: 42% and Group 3: 40% vs. Group 2: 21% and Group 4: 18%, *p*<0.05). Miscarriage rate was significantly higher in Group 4 (42% and 46% vs. 5%, 25% and 5% in Groups 1, 2 and 3, respectively, *p*<0.05).

**Conclusions:**

Our results showed lower blastocyst formation rates from IVF when males had high levels of sperm DNA fragmentation. Higher miscarriage rates were also observed in couples with males aged 40+ years. These results reinforce the need to inform couples with male partners aged 40+ years about the potential risks inherent to fertility treatment.

## INTRODUCTION

Advanced maternal age (>35 years) is known to be associated with progressive decreases in fertility and occurrence of embryonic chromosomal alterations ^(Leader *et al*., 2018)^. However, the influence of male age on reproductive outcomes has been largely ignored. Nonetheless, in recent years, the influence of the paternal component on embryo quality and success in achieving pregnancy both in natural pregnancies and IVF treatments has gained more attention ^(Kumar *et al*., 2013)^. Furthermore, it has been suggested that several parameters of embryo quality are strongly influenced by sperm quality, with sperm DNA fragmentation often being the main factor ^(Colaco & Sakkas, 2018)^. Sperm DNA fragmentation has been strongly linked to paternal age ^(Wyrobek *et al*., 2006)^. Some studies showed that DNA sperm repairing mechanisms are altered in males with advanced age, thereby inducing increases in the number of sperm with altered DNA ^(Muratori *et al*., 2019)^.

Studies showing decreases in male fertility potential after the age of 40 have recently reported that this age group accounts for more than 25% of males who start highly complex treatments with their partners worldwide ^(Evenson *et al*., 2020; Kaarouch *et al*., 2018; Stone *et al*., 2013)^. In addition, some studies have suggested that sperm DNA fragmentation levels are linked to poor embryo quality, low blastocyst development rates, higher global aneuploidy rates, low implantation rates, and recurrent miscarriages ^(Borges *et al*., 2019; Colaco & Sakkas, 2018)^.

Different studies support the tagging of semen samples as altered when they show >20% sperm DNA fragmentation ^(Agarwal *et al*., 2016; Van Montfoort *et al*., 2004; Zini *et al*., 2008)^, while other authors have propose >30% of affected sperm as a parameter ^(Colaco & Sakkas, 2018)^. These cutoff values depend on the sperm DNA fragmentation detection technique applied ^(Sakkas & Alvarez, 2010)^.

Based on the above, the aim of this study was to investigate the effects of sperm DNA fragmentation according to different paternal age groups in couples comprised of normozoospermic men and infertile women undergoing conventional IVF.

## MATERIALS AND METHODS

This retrospective study analyzed the outcomes of 163 couples submitted to IVF treatments at the Fertilis Reproductive Medicine in Buenos Aires, Argentina, between January 2019 and April 2020. The Institutional Ethics Committee approved the study protocol.

The inclusion criteria were as follows:

- Fresh transfer of embryos on Day 5 of embryo culture
- Women aged between 30 and 37 years; mature oocytes (MII): ≥4;
- Men meeting the following WHO sperm parameters (WHO, 2010):
  Volume: ≥1.5 mL
  Concentration: ≥15 x 10^6^ sperm/mL
  Progressive motility: ≥32%
  Viability: ≥58%
  Morphology: ≥4%

The males included in the study were aged between 28 and 55 years. Individuals with azoospermia, cryptozoospermia, retrograde ejaculation, leukocytospermia, or varicocele; subjects submitted to chemotherapy and radiation therapy; patients exposed to pesticides and other toxic agents; and men with a history of infection or fever in the three months prior to treatment were excluded. Cases in which, for some reason, IVF had to be performed by means of intracytoplasmic sperm injection (ICSI) or using frozen semen samples were also excluded.

Women with uterine factor, reproductive tract infection or disease, anovulation, or premature ovarian failure were excluded.

Semen samples were collected by masturbation in a sterile bottle after 2 to 5 days of sexual abstinence. Semen volume was measured after 30-60 minutes and semen concentration and motility were evaluated using a Makler counting chamber (Sefi Medical Instruments, Haifa, Israel). Next, sperm morphology was evaluated according to Kruger’s Strict Criteria ^(Kruger *et al*., 1986)^. To this end, a 5-10 µL semen aliquot was placed on a slide and a smear was performed and allowed to air dry. The slide was then submerged in 96% alcohol for 20 min for fixation, and immersed in Giemsa staining for 10 min. Then the slide was washed with water and allowed to dry at room temperature.

Sperm DNA fragmentation levels were determined by means of the Terminal Deoxynucleotidyl Transferase dUTP Nick End Labeling (TUNEL) assay ^(Lopes *et al*., 1998)^. To this end, Teflon Printed Slides for TUNEL (EMS, USA) were submerged for at least 2 h in Poly-L-Lysine 0.1% (Sigma, USA), and then rinsed with ultrapure water (Sigma, USA) and dried at room temperature. Semen samples were processed in a 15-ml centrifuge tube containing two layers of Pure Ception (SAGE, USA) at 90% and 50% ^(WHO, 2010)^. The samples were centrifuged at 300 g for 20 min, washed at 300 g for 10 min and re-suspended in 0.4 ml of heated human tubal fluid medium supplemented with 0.3% Human Albumin (SAGE, USA). Then the samples selected for this study were fixed with 37% formaldehyde (Sigma, USA) and stored at 4-8°C until use.

To evaluate DNA fragmentation, 30 µL aliquots of the samples were placed on excavated slides in duplicate. The slides were then placed in a wet chamber for 24h at 4-8°C. After that, the samples were washed three times for 5 min with 10 µL of phosphate buffered saline (PBS) 1X (Sigma, USA). Methanol (Sigma, USA) was then added for 90 sec and the slides were washed again three times with PBS 1X. Then, 10 µL of blocking solution (PBS + 0.5% Bovine Serum Albumin (BSA) (Sigma, USA) was added and allowed to act for 45 min inside the wet chamber at 4-8°C. Subsequently, another three washes with PBS 1X were performed.

**Figure 1:**
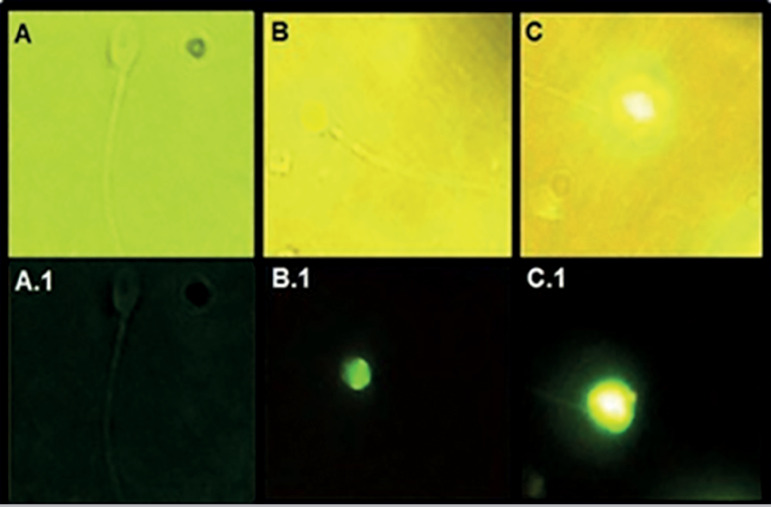
Spermatozoa with different TUNEL staining patterns (1000x) A: Negative sperm in white light; A.1: negative sperm with 0% fluorescence under UV light. B: Negative sperm with <50% fluorescence in white light; B.1: Negative sperm with <50% fluorescence in UV light. C: Positive sperm with >50% fluorescence in white light; C.1: Positive sperm with >50% fluorescence in UV light (from Riva et al., 2018)

A mixture of 30 µL of the fluorescent label and 5 µL of enzyme (In situ Death Cell Detection kit, Roche, USA) were added to each well of the excavated slides protected from light. Then the slides were placed in a wet chamber for 1 h on a thermal plate at 37°C. Next, three 5-min washes were performed with 10 µL of PBS 1X and the slides were allowed to dry completely at room temperature, always avoiding exposure to light. Finally, 5 µL of Vecta-Shield mounting agent (Vector Lab, USA) were added to each well and a 24 x 50 mm slide cover slip was placed above each well. Then the slides were examined under a fluorescence microscope (Nikon, Eclipse 200, Japan) at 1000x magnification with immersion oil. Sperm with fluorescence greater than 50% in the cytoplasm were considered positive, whereas the rest were considered negative (Figure 1). One extra well of the excavated slide was incubated with DNAse (1 U/mL; Sigma, USA) for 30 minutes at 37°C as a positive control, and in another well the TUNEL solution was omitted as a negative control. The number of sperm with fragmented DNA was recorded as the average of two counts of 100 sperm each (total 200) and the percentage of cells with positive TUNEL was calculated. Semen samples with TUNEL levels ≤20% were considered normal, whereas those with TUNEL levels >20% were considered altered.

Male patients were divided into four groups according to their age and TUNEL staining pattern in their semen samples:

Group 1: patients aged ≤39 years old with normal TUNEL assays (≤20%)
Group 2: patients aged ≤39 years old with altered TUNEL assays (>20%)
Group 3: patients aged ≥40 years with normal TUNEL assays (≤20%)
Group 4: patients aged ≥40 years old with altered TUNEL assays (>20%)

All female patients were stimulated with recombinant follicle stimulating hormone (FSH) (Gonal-F, Merck-Serono, Germany) combined with human *menopausal gonadotropin hormone (hMG)* (Menopur, Ferring, Sweden). An initial dose of 150 to 300 international units (IU) of gonadotropins was administered for 5 days, with adjustments made based on ovarian response. Upon reaching an average follicular diameter of 14 mm or estrogen levels of 300 pg/mL, a daily dose of gonadotropin-releasing hormone (GnRh) antagonist (Cetrorrelix, Cetrotide NR, Merck-Serono, Germany) was administered until discharge of ovulation, for which a single dose of 10,000 IU of human chorionic gonadotropin (hCG) (Gonacor 5000, Ferring Pharmaceuticals, Switzerland) was administered 34-36 h prior to follicular aspiration.

After follicular aspiration, the oocytes were place in Quinn’s Advantage protein plus Fertilization medium (SAGE, USA). The embryos produced after conventional IVF were grown until Day 3 in Quinn’s Advantage protein plus Cleavage medium (SAGE, USA), and were then placed in Quinn’s Advantage protein plus Blastocyst medium (SAGE, USA) until culture Day 5. The culture was carried out at 37°C in ESCO mini-Miri incubators in 5% oxygen and 6% carbon dioxide. Embryos from Day 5 were evaluated according to the Istanbul criteria (Alpha Scientists in Reproductive Medicine and ESHRE Special Interest Group of Embryology, 2011). The transfers were performed on culture Day 5, with only one embryo being transferred per patient using a Rocket-Echo Cath (Rocket Medical, England) catheter; the remaining embryos were cryopreserved. Clinical pregnancy was confirmed by ultrasound 6 weeks after embryo transfer.

The following parameters were determined: i) fertilization rate: MII oocytes fertilized relative to total inseminated MII oocytes; ii) blastocyst formation rate: embryos that reached the blastocyst stage relative to the number of fertilized oocytes; iii) expanded blastocyst formation rate: embryos that reached the expanded blastocyst stage relative to the number of fertilized oocytes; iv) clinical pregnancy rate: clinical pregnancies with fetal cardiac activity relative to total treatments; v) multiple pregnancy rate: pregnancies with more than one gestational sac relative to total pregnancies; vi) miscarriage rate: patients with pregnancies interrupted before 20 weeks of gestation relative to the total number of patients with a gestational sac; and vii) ongoing pregnancy rate: pregnancies ongoing after 20 weeks’ gestation relative to total number of patients treated. All parameters were defined according to the ICMART Glossary ^(Zegers-Hochschild *et al*., 2017)^.

### Statistical analysis

Statistical analysis was performed using GraphPad InStat 7 software (Graphpad Software Inc., San Diego, CA). The Kruskal Wallis test was used in the analysis of non-parametric data. Qualitative variables were analyzed with the Chi-square test. Statistical significance was set at <0.05.

## RESULTS

Groups 1 and 2 comprised male patients aged 34.9±4.2 years on average, whereas Groups 3 and 4 included male patients aged 45.1±3.7 years on average. DNA fragmentation levels measured using the TUNEL assay were 12.1±4.8% in groups with “normal fragmentation” (Groups 1 and 3) and 27.7±6.3% in groups with “altered fragmentation” (Groups 2 and 4).

Comparison of the study groups showed that semen characteristics such as viscosity, volume, concentration and vitality showed no significant differences (Table 1). In addition, female patients showed no significant differences in age, infertility factor distribution, progesterone level at the time of ovulation discharge, or endometrial thickness at the time of follicular aspiration (Table 2). Finally, analysis of the results of IVF treatments in the four groups showed no significant differences in total number of retrieved oocytes, number of mature oocytes (MII) retrieved, fertility rate or pregnancy and multiple pregnancy rates. In contrast, results showed significant differences in blastocyst formation rate and expanded blastocyst formation rate, both of which were lower in the two groups with DNA fragmentation levels >20% (Groups 2 and 4). Results also showed that the miscarriage rate in the group with males aged ≥40 years and altered DNA fragmentation levels (Group 4) was significantly higher; nevertheless, the ongoing pregnancy rate was not different between groups (Table 3).

**Table 1:** Comparison of the semen parameters of the four study groups.

	Group 1 Age ≤39 years normal DNA fragmentation	Group 2 Age ≤39 years altered DNA fragmentation	Group 3 Age ≥40 years normal DNA fragmentation	Group 4 Age ≥40 years altered DNA fragmentation
N	52	32	49	30
Mean male age	35.9±2.8^a^	35.7±2.6^a^	45.6±3.9^b^	46.4±3.4^b^
Normal viscosity	30/52 (58%)	16/32 (50%)	27/49 (55%)	16/30 (53%)
Volume (ml)	3.1±1.8	3.4±1.8	2.9±1.9	2.8±1.9
Concentration (10^6^spz/mL)*	62.3±55.5	73.6±52.1	69.7±52.0	63.7±56.2
Total count (10^6^ spz)*	192.5±143.7	232.4±161.1	220.9±196.3	195.6±163.8
Progressive spermatozoa (%)	44.9±19.9	44.6±16.5	45.2±21.9	45.9±23.2
Vitality (%)	74.1±11.0	73.8±10.1	74.5±13.2	71.9±15.1
Normal sperm morphology (%)	7.5±4.9	6.6±4.7	6.8±4.7	7.0±4.5
Concentration after processing (10^6^spz/mL)*	65.7±30.3	67.2±32.6	65.5±38.2	67.9±31.8
Motility after processing (%)	92.9±5.3	93.1±4.1	93.9±5.2	92.9±6.1
Total count after processing (10^6^spz)*	25.1±11.9	24.9±12.2	24.8±11.6	24.7±11.3

* spz = spermatozoa.

a,b Values with different letters inside the line differ significantly (*p*<0.05).

**Table 2:** Comparison of female patient characteristics in the four study groups.

	Group 1 Age ≤39 years normal DNA fragmentation	Group 2 Age ≤39 years altered DNA fragmentation	Group 3 Age ≥40 years normal DNA fragmentation	Group 4 Age ≥40 years altered DNA fragmentation
N	52	32	49	30
Female age	33.6±2.7	34.7±2.4	34.5±4.3	33.5±3.0
Tubal disconnection	23/52 (44%)	17/32 (53%)	25/49 (51%)	18/30 (60%)
Salpingectomy	23/52 (44%)	13/32 (41%)	21/49 (43%)	12/30 (40%)
Tubal ligation	5/52 (10%)	1/32 (3%)	2/49 (4%)	0/30 (0%)
Cervical factor	1/52 (2%)	1/32 (3%)	1/49 (2%)	0/30 (0%)
Progesterone (pg/mL)	0.5±0.3	0.5±0.2	0.5±0.3	0.5±0.2
Endometrium (mm)	8.0±2.4	8.0±2.2	7.9±2.7	7.9±2.5

**Table 3:** Comparison of IVF results obtained in the four study groups.

	Group 1 Age ≤39 years normal DNA fragmentation	Group 2 Age ≤39 years altered DNA fragmentation	Group 3 Age ≥40 years normal DNA fragmentation	Group 4 Age ≥40 years altered DNA fragmentation
N	52	32	49	30
Number of oocytes retrieved	8.3±3.8	8.2±4.3	7.8±3.5	8.0±3.9
Number of mature oocytes retrieved (MII)	6.2±2.4	6.0±3.2	5.9±4.0	6.0±3.3
Fertilization rate	283/321 (88%)	165/194 (85%)	24/287 (86%)	150/182 (82%)
Blastocyst formation rate	178/283 (63%)^a^	83/194 (43%)^b^	172/287 (60%)^a^	75/182 (41%)^b^
Expanded blastocyst formation rate	119/283 (42%)^a^	41/194 (21%)^b^	115/287 (40%)^a^	33/182 (18%)^b^
Clinical pregnancy rate	22/52 (42%)	12/32 (38%)	19/49 (39%)	11/30 (37%)
Multiple pregnancy rate	1/52 (1%)	0/32 (0%)	0/49 (0%)	0/30 (0%)
Miscarriage rate	1/22 (5%)^a^	3/12 (25%)^b^	1/19 (5%)^a^	5/11 (46%)^b^
Ongoing pregnancy rate	21/52 (40%)	9/32 (28%)	18/49 (37%)	6/30 (20%)

a,b Values with different letters inside the line differ significantly (*p*<0.05).

## DISCUSSION

Although some authors have previously reported a correlation between advanced paternal age and alteration in conventional semen parameters ^(Aitken *et al*., 2009; Alshahrani *et al*., 2014)^, in our study we found no significant differences in these semen parameters between males aged ≤39 years and males aged ≥40 years. However, since other types of alterations should not be underestimated, we evaluated the effect of sperm DNA fragmentation and found that increased levels of sperm DNA fragmentation (>20%) led to significant deterioration of IVF treatment outcomes in terms of the percentage of embryos that reached the blastocyst stage and embryos that reached the expanded blastocyst stage. This benefits groups with low sperm DNA fragmentation, as they will have higher cumulative pregnancy rates.

We also observed a marked effect of the combination of advanced male age and altered sperm DNA fragmentation on miscarriage rates, with groups meeting this description showing a miscarriage rate of 46% as compared with the other three groups with rates ranging between 5 and 25%. These findings are consistent with numerous studies that have reported a positive correlation between increased male age and sperm DNA damage ^(Agarwal *et al*., 2008; Johnson *et al*., 2011; Nijs *et al*., 2011; Ramasamy *et al*., 2015; Sharma *et al*., 2015)^. Nevertheless, ongoing pregnancy rate was not significant among groups. We believe that this might be due to the limited number of cases analyzed. We are currently recording pregnancy data from patients with failed first transfer attempts now undergoing transfers for the second time. Our hypothesis is that individuals with lower sperm DNA fragmentation might reach higher cumulative pregnancy rates. We would also like to explore in further studies the take-home baby rate, and it may be interesting to analyze the possible effect of fragmentation on the offspring.

There are several techniques for sperm DNA fragmentation analysis, including the Comet assay, SCD (Sperm Chromatin Dispersion), SCSA (Sperm Chromatin Structure Assay) and the TUNEL assay. We used the TUNEL assay in our study because previous reports had shown a high correlation between test results and pregnancy rates, yielding a high predictive value ^(Alvarez & Lewis, 2008; Borini *et al*., 2006; Greco *et al*., 2005)^. In addition, we used a cutoff point of 20% based on previous reports in which this threshold for TUNEL assays distinguished between fertile controls and infertile men, with high specificity and sensitivity ^(Sergerie *et al*., 2005)^.

It is known that sperm provides 50% of the embryo’s genome, thus making it vitally important for embryo development. The new embryo’s genome begins to express on Day 3 and, until that moment, development is almost exclusively dependent on the oocyte, while sperm acts only as a trigger in the process ^(Ortega *et al*., 2018)^. Until recently, the influence of male age on reproductive outcomes had been largely ignored. However, in recent years, the influence of the paternal component on embryo quality and on the success in achieving pregnancy both in natural pregnancies and IVF treatments has gained greater attention ^(Kumar *et al*., 2013)^.

In order to further the understanding of the correlation between sperm DNA fragmentation and IVF results, it is important to address the mechanisms primarily linked to sperm DNA damage. As some authors reported, there are three main mechanisms that cause DNA fragmentation: apoptosis induction, increased production of reactive oxygen species (ROS), and impairment of sperm chromatin maturation. These mechanisms can be induced by a variety of factors such as lifestyle, drugs, disease, aging, exposure to pollutants, and infection ^(Muratori *et al*., 2019; Sakkas & Alvarez, 2010)^. Although we attempted to exclude all such factors in patient selection, we have to consider other hidden variables impossible to study, such as epigenetics and metabolic effects that sperm DNA fragmentation might cause ^(Colaco & Sakkas, 2018)^.

Some authors have described a possible mechanism of sperm DNA damage repair by the oocyte during the fertilization stage or in later stages of embryo development, which might lead to the development of mosaic embryos ^(Jaroudi *et al*., 2009; Kaarouch *et al.,* 2015)^. A possible explanation for this phenomenon in male patients aged 40+ years is that this mechanism does not seem to be sufficient to repair sperm damage, and damage may be regarded as either very extensive or remain undetected with the tests carried out in our study. Thus, this may cause significant increases in miscarriage rates in this group of patients. Several studies found that sperm DNA fragmentation test values did not always correlate with pregnancy rate. Some authors believe that this is related to the type of DNA fragmentation mechanism in the sample. In some cases damage can be repaired by the oocyte, while in others, it cannot. Therefore, two patients might have the same DNA fragmentation value by the same test, with however different prognoses ^(Sakkas & Alvarez, 2010)^. Other authors believe that repair failures in sperm DNA damage may lead to *de novo* mutations and structural chromosomal alterations in the germline of male patients of advanced age, and that these mutations are eventually transmitted to the embryos ^(Beal *et al*., 2017)^. This might explain the increased miscarriage rate in this group of patients ^(Priskorn *et al*., 2014)^.

It is important to consider DNA fragmentation in sperm samples due to the fact that ICSI, a widely used technique, may facilitate the entry of damaged sperm into the oocyte, since having normal morphology does not necessarily correlate with absence of DNA damage. It is relevant to identify the male factors that affect embryo quality, considering that when they are severe, there is an increased risk of transmitting genetic disorders to the offspring ^(Colaco & Sakkas, 2018)^. Furthermore, recent studies have assessed the correlation between paternal age and increased risk of autism onset in born children ^(Sandin *et al*., 2016)^. These studies have reported that, for the general population, the rate of autism among children born to male parents over the age of 50 is 66% higher than that of children born to 20-year-old fathers. In addition, the rate of autism in children born to male parents aged between 40 and 49 years was 28% higher than that of children born to 20-year-old fathers. These results, together with the findings reported in the present study, clearly show the need to inform couples with males aged 40+ years seeking fertility treatment of these potential risks.

One way to decrease sperm DNA damage is to perform lifestyle changes such as avoiding smoking, pollutants, contaminants, and factors that increase oxidative stress ^(Cui *et al*., 2016; Henkel & Franken, 2011; Rybar *et al*., 2011; Wright *et al*., 2014)^. These might help increase the reproductive success rates in this group of patients. However, a reliable method to treat semen samples with high levels of DNA fragmentation should be identified, since the methods in use have not clearly demonstrated their reliability ^(Cakar *et al*., 2016; Nadalini *et al*., 2014; Romany *et al*., 2014; Tavalaee *et al*., 2012)^. In this regard, a microfluidic system that has been recently developed is showing encouraging results, and seems, to this point, to be the most suitable alternative for the treatment of this type of sperm condition ^(Nosrati *et al*., 2017; Parrella *et al*., 2019; Quinn *et al*., 2018; Shirota *et al*., 2016)^. This system uses a chip made of polymethylmethacrylate and has a microfilter that only allows sperm with low DNA fragmentation levels and with the highest progressive motility to pass. This allows collecting sperm samples with probably lower percentages of DNA damage, thereby increasing the chances of obtaining good quality embryos ^(Samuel *et al*., 2018; Yetkinel *et al*., 2019)^. Given the results of this study, we have decided to introduce this technique in order to remove affected sperm in patients with high DNA fragmentation values.

Our results revealed a significant effect of advanced paternal age on IVF results, making it necessary to study sperm DNA fragmentation levels in male patients willing to undergo fertility treatment.

## References

[r1] Agarwal A, Makker K, Sharma R (2008). Clinical relevance of oxidative stress in male factor infertility: an update. Am J Reprod Immunol..

[r2] Agarwal A, Majzoub A, Esteves SC, Ko E, Ramasamy R, Zini A (2016). Clinical utility of sperm DNA fragmentation testing: practice recommendations based on clinical scenarios. Transl Androl Urol.

[r3] Aitken RJ, De Iuliis GN, McLachlan RI (2009). Biological and clinical significance of DNA damage in the male germ line. Int J Androl..

[r4] Alpha Scientists in Reproductive Medicine and ESHRE Special Interest Group of Embryology (2011). The Istanbul consensus workshop on embryo assessment: proceedings of an expert meeting. Hum Reprod..

[r5] Alshahrani S, Agarwal A, Assidi M, Abuzenadah AM, Durairajanayagam D, Ayaz A, Sharma R, Sabanegh E (2014). Infertile men older than 40 years are at higher risk of sperm DNA damage. Reprod Biol Endocrinol..

[r6] Alvarez JG, Lewis S (2008). Sperm chromatin structure assay parameters measured after density gradient centrifugation are not predictive of the outcome of ART. Hum Reprod..

[r7] Beal MA, Yauk CL, Marchetti F

[r8] Borges E Jr, Zanetti BF, Setti AS, Braga DPAF, Provenza RR, Jr. Iaconelli A (2019). Sperm DNA fragmentation is correlated with poor embryo development, lower implantation rate, and higher miscarriage rate in reproductive cycles of non-male factor infertility. Fertil Steril.

[r9] Borini A, Tarozzi N, Bizzaro D, Bonu MA, Fava L, Flamigni C, Coticchio G (2006). Sperm DNA fragmentation: paternal effect on early post-implantation embryo development in ART. Hum Reprod..

[r10] Cakar Z, Cetinkaya B, Aras D, Koca B, Ozkavukcu S, Kaplanoglu İ, Can A, Cinar O (2016). Does combining magnetic-activated cell sorting with density gradient or swim-up improve sperm selection?. J Assist Reprod Genet..

[r11] Colaco S, Sakkas D (2018). Paternal factors contributing to embryo quality. J Assist Reprod Genet..

[r12] Cui X, Jing X, Wu X, Wang Z, Li Q (2016). Potential effect of smoking on semen quality through DNA damage and the downregulation of Chk1 in sperm. Mol Med Rep..

[r13] Evenson DP, Djira G, Kasperson K, Christianson J (2020). Relationships between the age of 25,445 men attending infertility clinics and sperm chromatin structure assay (SCSA®) defined sperm DNA and chromatin integrity. Fertil Steril.

[r14] Greco E, Scarselli F, Iacobelli M, Rienzi L, Ubaldi F, Ferrero S, Franco G, Anniballo N, Mendoza C, Tesarik J (2005). Efficient treatment of infertility due to sperm DNA damage by ICSI with testicular spermatozoa. Hum Reprod..

[r15] Henkel RR, Franken DR (2011). Sperm DNA fragmentation: origin and impact on human reproduction. J Reprod Biotechnol Fertil.

[r16] Jaroudi S, Kakourou G, Cawood S, Doshi A, Ranieri DM, Serhal P, Harper JC, SenGupta SB (2009). Expression profiling of DNA repair genes in human oocytes and blastocysts using microarrays. Hum Reprod..

[r17] Johnson GD, Lalancette C, Linnemann AK, Leduc F, Boissonneault G, Krawetz SA (2011). The sperm nucleus: chromatin, RNA, and the nuclear matrix. Reproduction.

[r18] Kaarouch I, Bouamoud N, Louanjli N, Madkour A, Copin H, Benkhalifa M, Sefrioui O (2015). Impact of sperm genome decay on Day-3 embryo chromosomal abnormalities from advanced-maternal-age patients. Mol Reprod Dev.

[r19] Kaarouch I, Bouamoud N, Madkour A, Louanjli N, Saadani B, Assou S, Aboulmaouahib S, Amzazi S, Copin H, Benkhalifa M, Sefrioui O (2018). Paternal age: Negative impact on sperm genome decays and IVF outcomes after 40 years. Mol Reprod Dev..

[r20] Kruger TF, Menkveld R, Stander FS, Lombard CJ, Van der Merwe JP, van Zyl JA, Smith K (1986). Sperm morphologic features as a prognostic factor in in vitro fertilization. Fertil Steril.

[r21] Kumar M, Kumar K, Jain S, Hassan T, Dada R (2013). Novel insights into the genetic and epigenetic paternal contribution to the human embryo. Clinics.

[r22] Leader J, Bajwa A, Lanes A, Hua X, Rennicks White R, Rybak N, Walker M (2018). The Effect of Very Advanced Maternal Age on Maternal and Neonatal Outcomes: A Systematic Review. J Obstet Gynaecol Can.

[r23] Lopes S, Jurisicova A, Sun JG, Casper RF (1998). Reactive oxygen species: potential cause for DNA fragmentation in human spermatozoa. Hum Reprod..

[r24] Muratori M, Marchiani S, Tamburrino L, Baldi E (2019). Sperm DNA Fragmentation: Mechanisms of Origin. Adv Exp Med Biol..

[r25] Nadalini M, Tarozzi N, Di Santo M, Borini A (2014). Annexin V magnetic-activated cell sorting versus swim-up for the selection of human sperm in ART: is the new approach better then the traditional one?. J Assist Reprod Genet.

[r26] Nijs M, De Jonge C, Cox A, Janssen M, Bosmans E, Ombelet W (2011). Correlation between male age, WHO sperm parameters, DNA fragmentation, chromatin packaging and outcome in assisted reproduction technology. Andrologia.

[r27] Nosrati R, Graham PJ, Zhang B, Riordon J, Lagunov A, Hannam TG, Escobedo C, Jarvi K, Sinton D (2017). Microfluidics for sperm analysis and selection. Nat Rev Urol.

[r28] Ortega NM, Winblad N, Plaza Reyes A, Lanner F (2018). Functional genetics of early human development. Curr Opin Genet Dev.

[r29] Parrella A, Keating D, Cheung S, Xie P, Stewart JD, Rosenwaks Z, Palermo GD (2019). A treatment approach for couples with disrupted sperm DNA integrity and recurrent ART failure. J Assist Reprod Genet.

[r30] Priskorn L, Jensen TK, Lindahl-Jacobsen R, Skakkebæk NE, Bostofte E, Eisenberg ML (2014). Parental age at delivery and a man’s semen quality. Hum Reprod..

[r31] Quinn MM, Jalalian L, Ribeiro S, Ona K, Demirci U, Cedars MI, Rosen MP (2018). Microfluidic sorting selects sperm for clinical use with reduced DNA damage compared to density gradient centrifugation with swim-up in split semen samples. Hum Reprod..

[r32] Ramasamy R, Scovell JM, Kovac JR, Cook PJ, Lamb DJ, Lipshultz LI (2015). Fluorescence in situ hybridization detects increased sperm aneuploidy in men with recurrent pregnancy loss. Fertil Steril.

[r33] Riva NS, Ruhlmann C, Iaizzo RS, Marcial López CA, Martínez AG (2018). Comparative analysis between slow freezing and ultra-rapid freezing for human sperm cryopreservation. JBRA Assist Reprod.

[r34] Romany L, Garrido N, Motato Y, Aparicio B, Remohí J, Meseguer M (2014). Removal of annexin V-positive sperm cells for intracytoplasmic sperm injection in ovum donation cycles does not improve reproductive outcome: a controlled and randomized trial in unselected males. Fertil Steril.

[r35] Rybar R, Kopecka V, Prinosilova P, Markova P, Rubes J (2011). Male obesity and age in relationship to semen parameters and sperm chromatin integrity. Andrologia.

[r36] Sakkas D, Alvarez JG (2010). Sperm DNA fragmentation: mechanisms of origin, impact on reproductive outcome, and analysis. Fertil Steril.

[r37] Samuel R, Feng H, Jafek A, Despain D, Jenkins T, Gale B (2018). Microfluidic-based sperm sorting & analysis for treatment of male infertility. Transl Androl Urol.

[r38] Sandin S, Schendel D, Magnusson P, Hultman C, Surén P, Susser E, Grønborg T, Gissler M, Gunnes N, Gross R, Henning M, Bresnahan M, Sourander A, Hornig M, Carter K, Francis R, Parner E, Leonard H, Rosanoff M, Stoltenberg C (2016). Autism risk associated with parental age and with increasing difference in age between the parents. Mol Psychiatry.

[r39] Sergerie M, Laforest G, Bujan L, Bissonnette F, Bleau G (2005). Sperm DNA fragmentation: threshold value in male fertility. Hum Reprod..

[r40] Sharma R, Agarwal A, Rohra VK, Assidi M, Abu-Elmagd M, Turki RF (2015). Effects of increased paternal age on sperm quality, reproductive outcome and associated epigenetic risks to offspring. Reprod Biol Endocrinol..

[r41] Shirota K, Yotsumoto F, Itoh H, Obama H, Hidaka N, Nakajima K, Miyamoto S (2016). Separation efficiency of a microfluidic sperm sorter to minimize sperm DNA damage. Fertil Steril.

[r42] Stone BA, Alex A, Werlin LB, Marrs RP (2013). Age thresholds for changes in semen parameters in men. Fertil Steril.

[r43] Tavalaee M, Deemeh MR, Arbabian M, Nasr-Esfahani MH (2012). Density gradient centrifugation before or after magnetic-activated cell sorting: which technique is more useful for clinical sperm selection?. J Assist Reprod Genet.

[r44] Van Montfoort AP, Dumoulin JC, Kester AD, Evers JL (2004). Early cleavage is a valuable addition to existing embryo selection parameters: a study using single embryo transfers. Hum Reprod..

[r45] World Health Organization (WHO) (2010). WHO Laboratory Manual for the Examination and Processing of Human Semen.

[r46] Wright C, Milne S, Leeson H (2014). Sperm DNA damage caused by oxidative stress: modifiable clinical, lifestyle and nutritional factors in male infertility. Reprod Biomed Online.

[r47] Wyrobek AJ, Eskenazi B, Young S, Arnheim N, Tiemann-Boege I, Jabs EW, Glaser RL, Pearson FS, Evenson D (2006). Advancing age has differential effects on DNA damage, chromatin integrity, gene mutations, and aneuploidies in sperm. Proc Natl Acad Sci USA.

[r48] Yetkinel S, Kilicdag EB, Aytac PC, Haydardedeoglu B, Simsek E, Cok T (2019). Effects of the microfluidic chip technique in sperm selection for intracytoplasmic sperm injection for unexplained infertility: a prospective, randomized controlled trial. J Assist Reprod Genet.

[r49] Zegers-Hochschild F, Adamson GD, Dyer S, Racowsky C, de Mouzon J, Sokol R, Rienzi L, Sunde A, Schmidt L, Cooke ID, Simpson JL, van der Poel S (2017). The International Glossary on Infertility and Fertility Care, 2017. Hum Reprod..

[r50] Zini A, Boman JM, Belzile E, Ciampi A (2008). Sperm DNA damage is associated with an increased risk of pregnancy loss after IVF and ICSI: systematic review and meta-analysis. Hum Reprod..

